# Gastrinoma and neurofibromatosis type 2: the first case report and review of the literature

**DOI:** 10.1186/1471-230X-14-110

**Published:** 2014-06-24

**Authors:** Sara Massironi, Alessandra Zilli, Roberta Elisa Rossi, Federica Cavalcoli, Dario Conte, Maddalena Peracchi

**Affiliations:** 1 Gastroenterology and Endoscopy Unit, Fondazione IRCCS Ca’ Granda - Ospedale Maggiore Policlinico, Università degli Studi di Milano, Via F. Sforza 35, Milano 20122, Italy; 2 Department of Pathophysiology and Organ Transplant, Fondazione IRCCS Ca’ Granda - Ospedale Maggiore Policlinico, Università degli Studi di Milano, Milan, Italy

**Keywords:** Gastrinoma, Neurofibromatosis type 2, Multiple endocrine neoplasia syndrome type 1, Neuroendocrine tumors, Zollinger Ellison syndrome

## Abstract

**Background:**

Gastroenteropancreatic neuroendocrine tumors have occasionally been described in association with neurofibromatosis type 1, whereas an association with neurofibromatosis type 2 has never been reported.

**Case presentation:**

This report refers to an Italian 69 year old woman with neurofibromatosis type 2 and a pancreatic gastrinoma. In the past she had encephalic meningiomas, a tongue schwannoma and bilateral acoustic neurinomas. She presented with weight loss and a long-term history of diarrhea, responsive to proton pump inhibitors. Upper gastrointestinal endoscopy revealed peptic ulcer of the duodenal bulb. Blood tests were normal, except for the elevation of plasma gastrin (1031 pg/ml; reference value <108) and chromogranin A (337 U/L; reference value <36). After secretin stimulation testing, the plasma gastrin level rose to 3789 pg/ml. The abdomen magnetic resonance imaging and gallium68-DOTATOC positron emission tomography scan demonstrated the presence of a 1.2 x 2 cm lesion in the pancreatic head and a liver metastatis. Pancreatic endoscopic ultrasound with fine needle aspiration revealed cytomorphologic features suggestive of pancreatic gastrinoma. Brain magnetic resonance showed a pituitary microadenoma. There was no evidence of hyperparathyroidism. The genetic test for multiple endocrine neoplasia type 1 syndrome mutation was negative.

**Conclusion:**

This report focuses on the first case of coexistence of gastrinoma with neurofibromatosis type 2. Although the clinical relevance of this association remains to be determined, our case report will surely give cause for due consideration.

## Background

Both gastroenteropancreatic neuroendocrine tumors (GEP NET) and neurofibromatosis type 2 (NF2) are fairly rare diseases [1,2]. GEP NET have occasionally been described in association with neurofibromatosis type 1 (NF1) [3], whereas an association with NF2 has never been reported.

Gastrinoma is a gastrin-secreting tumor usually located in the duodenal wall or in the pancreas [4]. Chronic hypergastrinemia causes gastric acid hypersecretion with consequent peptic ulcer disease, diarrhea and gastroesophageal reflux disease (Zollinger Ellison syndrome, ZES), usually responsive to proton pump inhibitor (PPI) therapy. With an incidence of 0.5-2/million population/year gastrinoma may be sporadic or may develop in the setting of multiple endocrine neoplasia type 1 (MEN1) syndrome [5-8]. The tumors associated with MEN1 syndrome have a more benign course than sporadic neuroendocrine tumors, although more aggressive tumors have rarely been described in association with MEN1 syndrome [9]. MEN1 is an autosomal dominant inherited disease, due to inactivating mutations of the MEN1 gene, located on the long arm of chromosome 11 (11q13) [10]. Diagnostic criteria for MEN1 include the presence of at least two of the three main MEN1 associated lesions (primary hyperparathyroidism, GEP NET and anterior pituitary tumors) or the association of any typical MEN1-associated neoplasia and a positive familial history [11]. Diagnostic confirmation is achievable through MEN1 genetic testing.

According to available data, an association between GEP NET and NF1 has already been reported and it is well recognized that NF1 patients may develop NET, usually in the duodenum or periampullary region [12-14] and occasionally in the pancreas [15]. Gastric carcinoids have also been described [16].

NF2 is an autosomal dominant inherited disease characterized by the predisposition to develop tumors of the central nervous system (CNS) [2]. NF2 is caused by mutations of the NF2 gene that codes for an oncosuppressor protein named merlin or schwannomin and is located on chromosome 22 (22q12.2) [17]. A common manifestation of the disease is the development of vestibular schwannomas, tumors in other cranial nerves and meningiomas, usually multifocal and intracranial, but sometimes also spinal or intradural [18].

At present an association between GEP NET and NF2 has never been reported. The present case report refers to a woman with NF2 and a pancreatic gastrinoma.

## Case presentation

**Figure 1:**
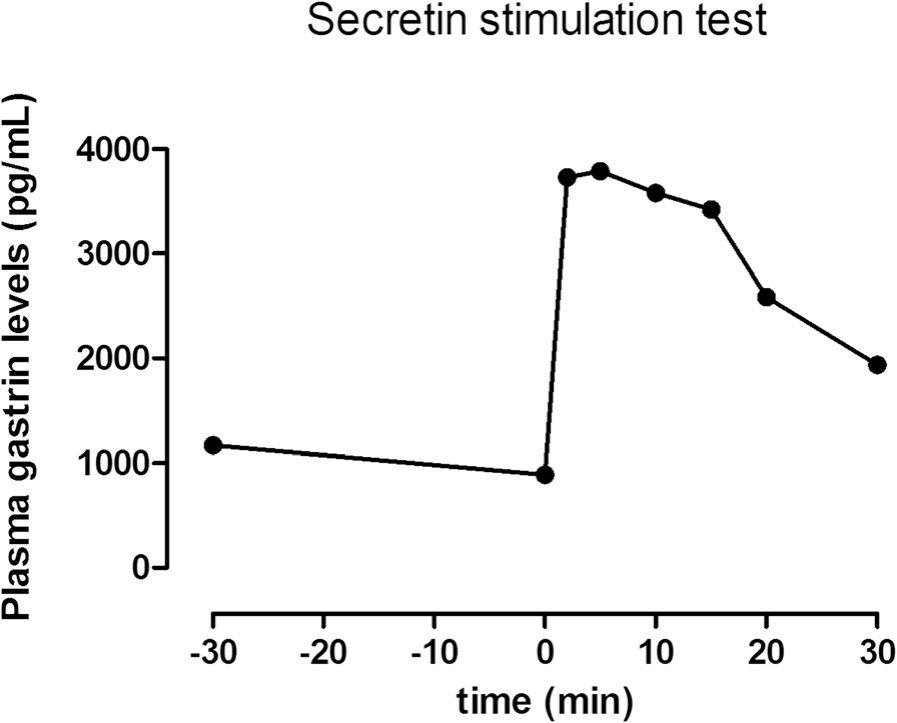
**Secretin stimulation test in the patient.** Plasma gastrin levels were measured at -30 and 0 time, and 2, 5, 10, 15, 20 and 30 minutes after intravenous secretin infusion (Secrelux 2U/kg, Goldham-Bioglan, Zusmarhausen. Germany). The increase from 1031 to 3789 pg/ml was observed within ten minutes.

**Figure 2:**
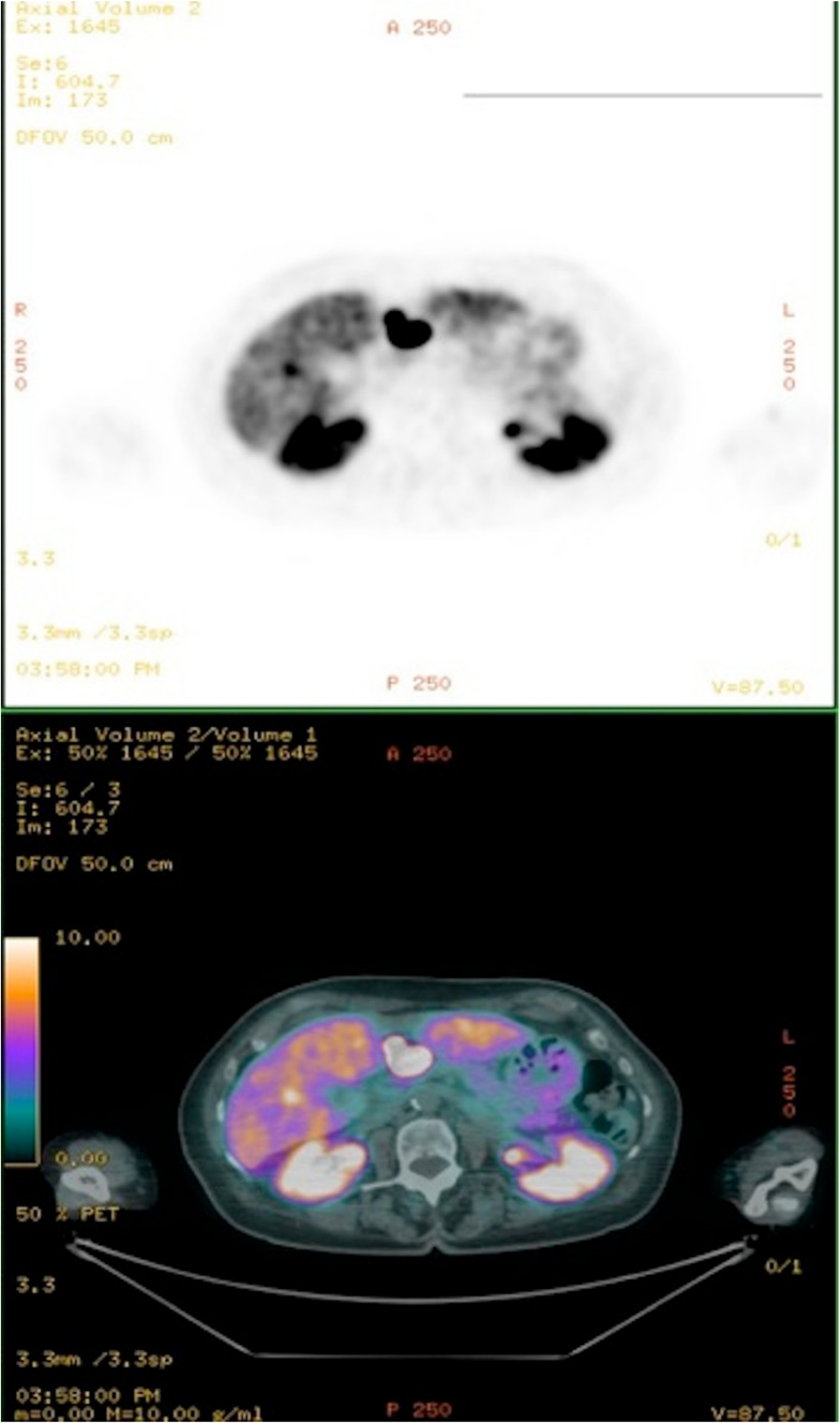
**Gallium-68-DOTATOC PET.** Gallium68 PET detected the presence of a nodular lesion of cm 1.2 x 2 in the pancreatic head and a suspected liver metastatic lesion.

**Figure 3:**
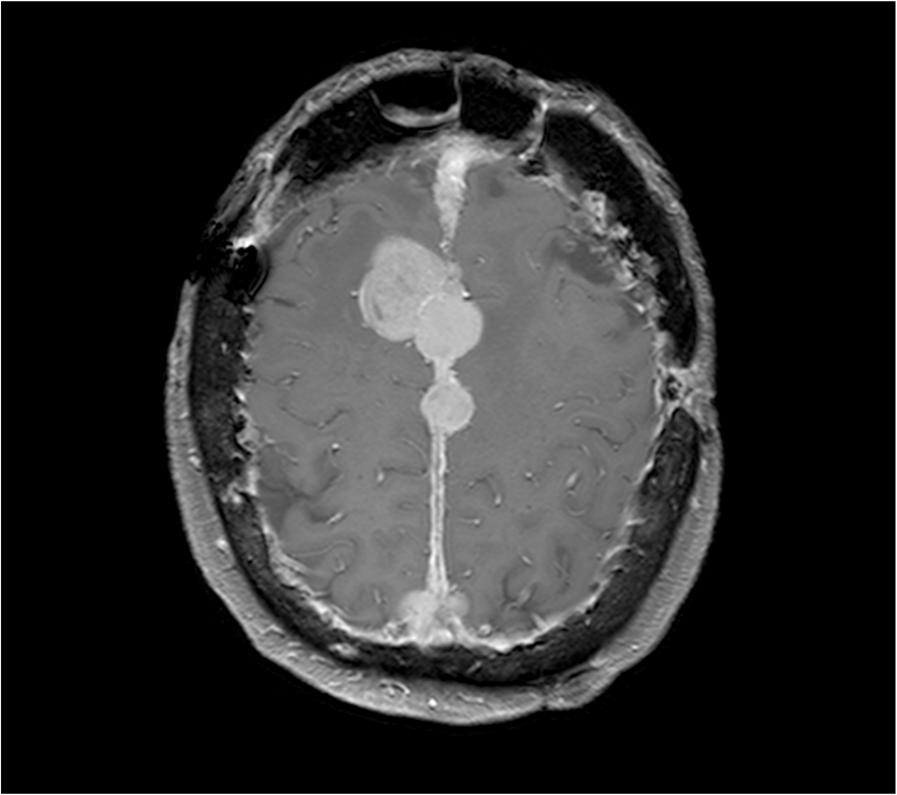
**Brain MR with contrast.** This T1-weighted image detected multiple meningiomas enhancing intensely and homogeneously after administration of gadolinium.

In 2011 an Italian 69 years old woman was referred to our Gastrointestinal Unit due to an 8 years long history of diarrhea, dyspepsia and weight loss. Diarrhea was responsive to PPI. Two years before (2009) she was diagnosed as having NF2 syndrome, according to internationally accepted clinical criteria [19], based on the finding of multiple encephalic meningiomas (partially removed by surgery), a tongue schwannoma and bilateral acoustic neurinomas. In addition, a non-functioning adrenal adenoma, has also been diagnosed. To investigate the cause of diarrhea, the patient had already undergone both a colonoscopy, negative, and an upper gastrointestinal endoscopy, which provided clear cut evidence of a duodenal bulb ulceration; during the endoscopy the measurement of intragastric pH was not performed, since the patient was on PPI. After two weeks of PPI withdrawal laboratory tests revealed a marked increase in plasma gastrin levels (1051 pg/ml; reference value, r.v. <108), chromogranin A (337 U/L; r.v. <36), glucagon (524 pg/ml; r.v. 25–250). The liver, kidney, thyroid and parathyroid function tests were normal. Moreover, secondary causes of hypergastrinemia were excluded.A secretin stimulation test (Secrelux 2U/Kg, Goldham-Bioglan, Zusmarhausen. Germany) was performed and showed a diagnostic increase of plasma gastrin levels (from 1031 to 3789 pg/ml) within ten minutes from the secretin injection (Figure 1). The patient underwent Octreoscan® with the evidence of a hypercaptant pancreatic lesion and a second one in the right frontal area, consistent with a meningiomatous lesion. Other imaging studies included abdomen magnetic resonance (MR) and Gallium-68-DOTATOC positron emission tomography (PET) (Figure 2), which revealed a 1.2 × 2 cm nodular lesion in the pancreatic head, a liver metastatis and, the already known cerebral meningioma. After several months the patient underwent pancreatic endoscopic ultrasound with fine needle aspiration, demonstrating cytomorphologic features suggestive of pancreatic gastrinoma, with well-differentiated cells expressing Chromogranin A, synaptophysin and gastrin at immunohistochemistry. Ki-67 index was 2% and no mitosis was found in the sample.Due to the coexistence of a gastrinoma and an adrenal adenoma, a brain and encephalic trunk MR (Figure 3) was performed to investigate the possible presence of a pituitary neoplasm: this revealed partial empty sella, the residual meningiomatous lesions and a pituitary microadenoma, which resulted non functioning at hormonal blood tests. There was no evidence of hyperparathyroidism, but, given the presence of a pancreatic gastrinoma, a pituitary and an adrenal adenoma, the patient underwent genetic testing for MEN1 mutations, which was negative. Genetic test for the CDKN1B gene was not performed since the patient died because of massive bleeding from a cerebral meningioma.

## Conclusions

We have reported the first case report of the association between NF2 and a pancreatic gastrinoma, although it remains to be determined whether this association is merely casual or not. The possible underlying pathogenetic mechanisms need also elucidation.

The association between NF1 and GEP NET has already been established. Several case reports and reviews of the literature describe the coexistence of NF1 and neuroendocrine neoplasms, such as somatostatinomas, gastrinomas [20], insulinomas [21] and gastric carcinoids [8-12]. In patients with NF1 NETs are most commonly localized in the duodenum and in the peri-ampullary region. Although NF1 associated duodenal neuroendocrine tumors are not distinctive, they tend to be pure somatostatinomas [22,23] or less frequently gastrinomas, whereas duodenal tumors not associated with NF1 are frequently multihormonal, but asymptomatic [24]. Other gastrointestinal manifestations can occur in several locations, in the form of gastrointestinal stromal tumors or ganglioneuromas [3]. Also an association between NF1 and MEN2A is described in one case report [25].

As concerns patients with NF2, schwannomas of the intestinal tract are a possible complication [26], whereas there are no data about coexistence with GEP NET and/or MEN1 syndrome.

An increased occurrence of endocrine tumors, as well as of other non endocrine tumors, including collagenomas, ependimomas, schwannomas and meningiomas has been recently reported in patients with MEN1 [27-29]. Meningiomas arising in MEN1 setting are usually silent, thus the diagnosis is often late, with a mean interval of 18 years from the diagnosis of MEN1; they have a higher incidence in patients with ZES in the MEN1 setting than in patients with sporadic ZES [30].

The present report focuses on the first case of gastrinoma in a NF2 patient. This patient might have a MEN1 like phenotype, as she showed the clinical features typical of MEN1 syndrome (pancreatic neuroendocrine tumor, pituitary microadenoma and adrenal adenoma), despite the absence of hyperparathyroidism and MEN1 gene mutations. The phenotype of MEN1 is broad and different combinations of endocrine and non endocrine manifestations have been described. About 30% MEN1 patients do not carry detectable MEN1 gene mutations [31]. Patients without detectable mutations may have mutations in introns or in the regulatory or untranslated regions. Moreover, the polymerase chain reaction does not reveal a large deletion causing the loss of a whole exon. Recently, germ line mutations have been found in the CDKN1B gene, encoding a cyclin dependent kinase inhibitor protein that inhibits cell cycle progression [32,33], even if only 1.6-2.8% of all tested cases of MEN1 have CDKN1B mutations [34]. The possible pathogenic mechanisms underlying the dual development of gastrinoma and NF2 remain to be elucidated. Interestingly, a possible explanation for the high prevalence of neuroendocrine tumors in NF1 might be the loss of neurofibromin, a tumor suppressor protein, representing the main product of the NF1 gene. Again, the mutation of the G protein Ras, often seen in NF1 tumors, could promote tumorigenesis due to a prolonged or persistent arrest in the activated GTP loaded state. Recent studies have clearly shown that also the tumor-suppressor protein merlin, mutated in NF2, controls Ras activity [35]. Furthermore, merlin probably regulates angiogenesis to support schwannoma growth, although the exact mechanisms are still unexplored [36]. Angiogenesis is an important determinant of tumor growth also in neuroendocrine neoplasms, so merlin could influence their development and growth. Another possible explanation of the association between NF2 and GEP NET may be the likely common embryological origin of nervous and neuroendocrine cells from the neural crest, although some authors have recently suggested that the neuroendocrine system might originate from the endoderm [37,38].

Overall, the possible pathogenic mechanisms underlying the occurrence of both gastrinoma and NF2, which would allow to discriminate between an actual connection and a merely casual association, remain to be elucidated.

## Consent

Written informed consent was obtained from the patient for publication of this case report and any accompanying images. A copy of the written consent is available for review by the Editor-in-Chief of this journal.

## Abbreviations

GEP NET: Gastroenteropancreatic neuroendocrine tumors; NF2: Neurofibromatosis type 2; NF1: Neurofibromatosis type 1; ZES: Zollinger Ellison syndrome; PPI: Proton pump inhibitor; MEN1: Multiple endocrine neoplasia type 1; CNS: Central nervous system; MR: Magnetic resonance; PET: Positron emission tomography.

## Competing interests

We have no conflict of interest to declare.

## Authors’ contributions

SM planned the work. SM, AZ and RER wrote the paper and subsequently performed its critical review, contributing equally to this work. AZ and FC carried out the literature research; MP contributed to the acquisition of data and their interpretation. DC and MP revised all the materials and manuscript. All authors read and approved the final manuscript.

## Pre-publication history

The pre-publication history for this paper can be accessed here:

http://www.biomedcentral.com/1471-230X/14/110/prepub
